# Supplementary figures and images for: A novel, ataxic mouse model of ataxia telangiectasia caused by a clinically relevant nonsense mutation
Source: eLife. 2021 Nov 1;10:e64695. doi: 10.7554/eLife.64695 (PMC8601662; doi:10.7554/eLife.64695)

Panel C, left

+/+    R35X/+    R35X/  
                         R35X

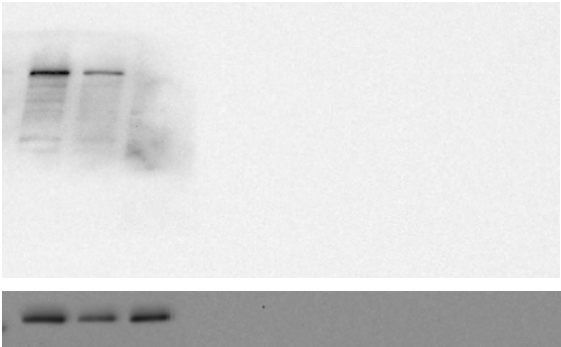

Panel C, right

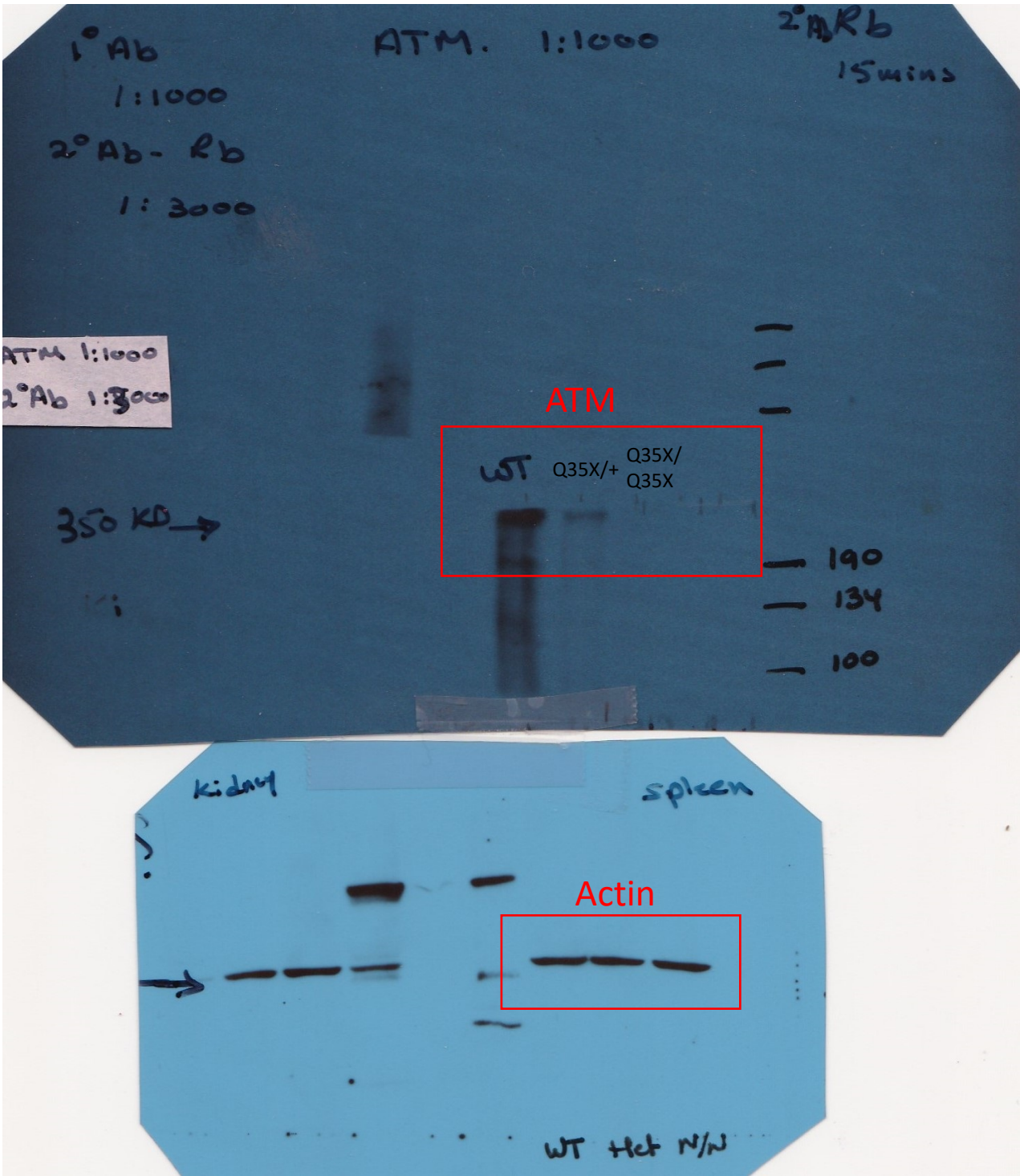

Supplement: Figure 1—source data 1. [file elife-64695-fig1-data1.zip › Fig.1C blots.pdf]

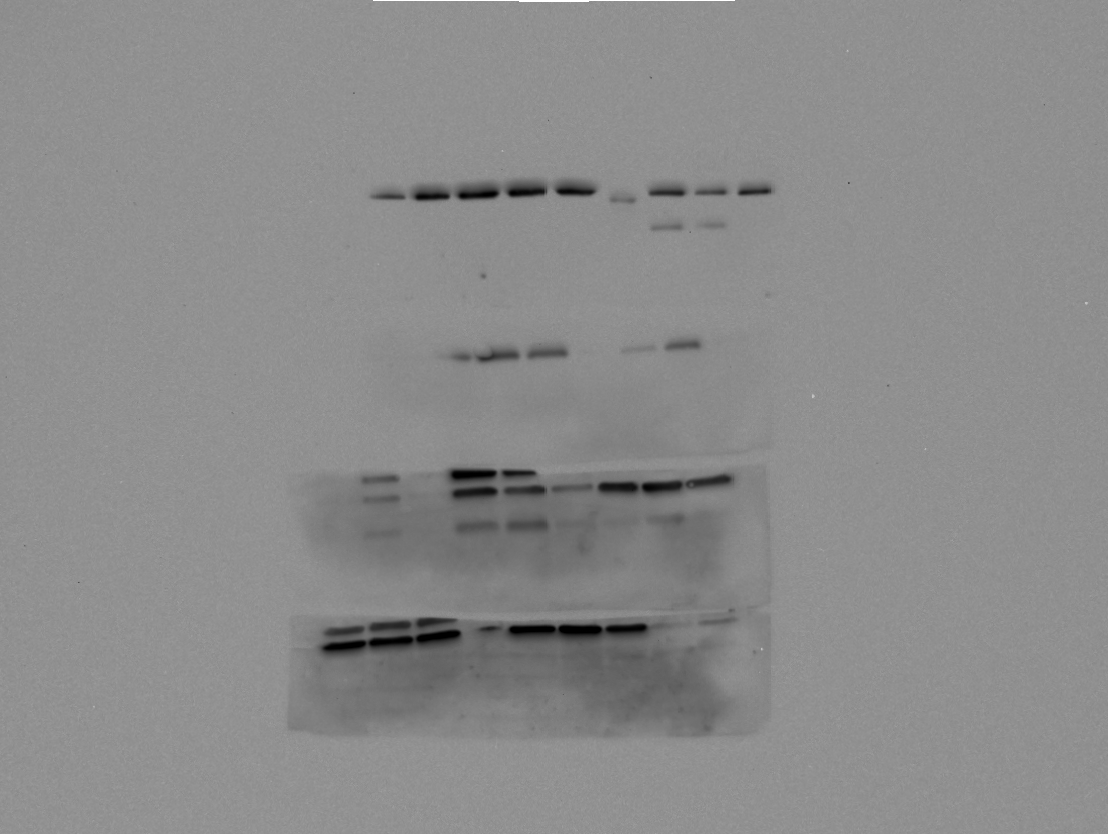

Supplement: Figure 1—source data 1. [file elife-64695-fig1-data1.zip › Fig.1PanelC-R35X-actin.jpg]

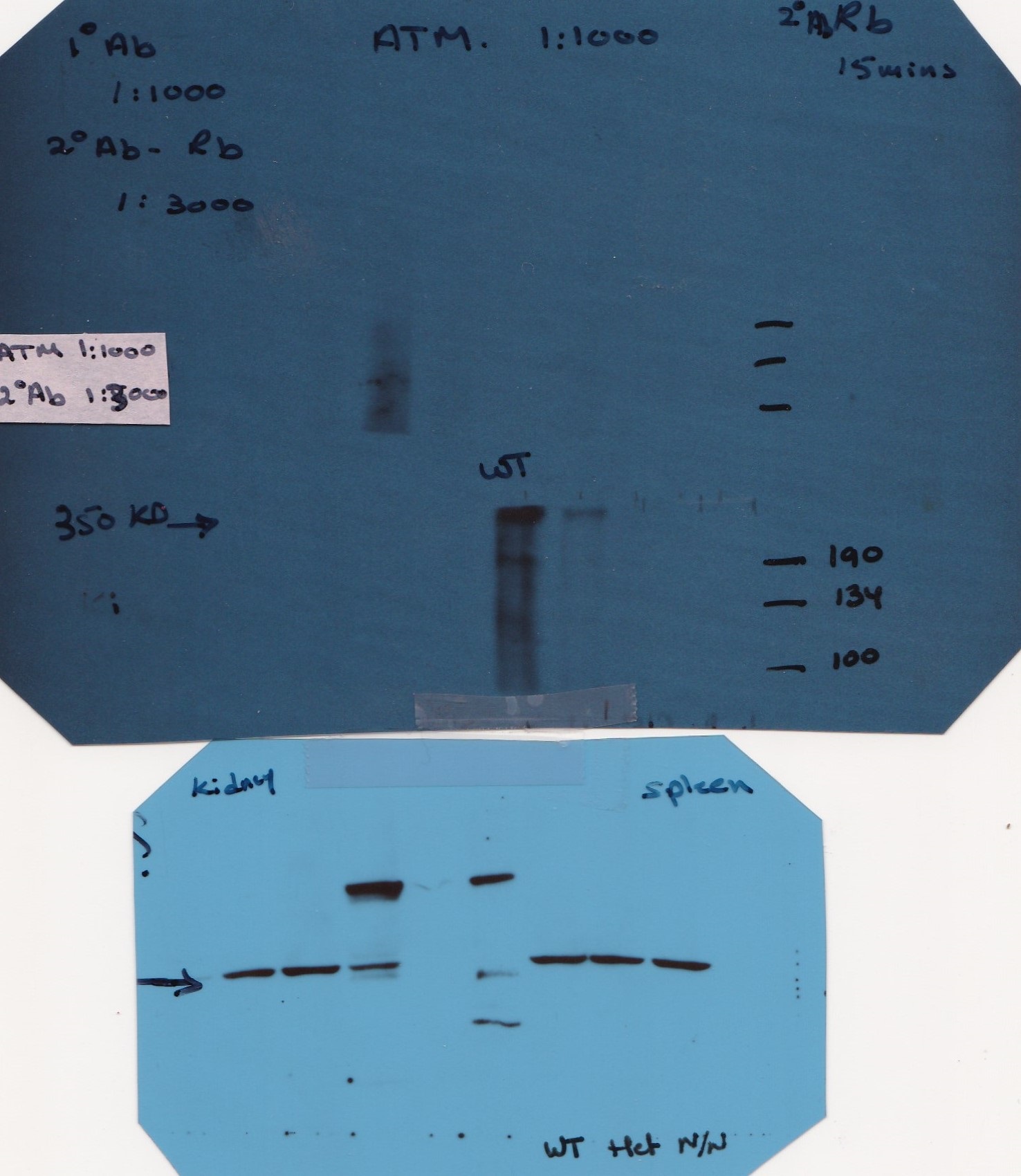

Supplement: Figure 1—source data 1. [file elife-64695-fig1-data1.zip › Fig.1_PanelC_Q35X.jpg]

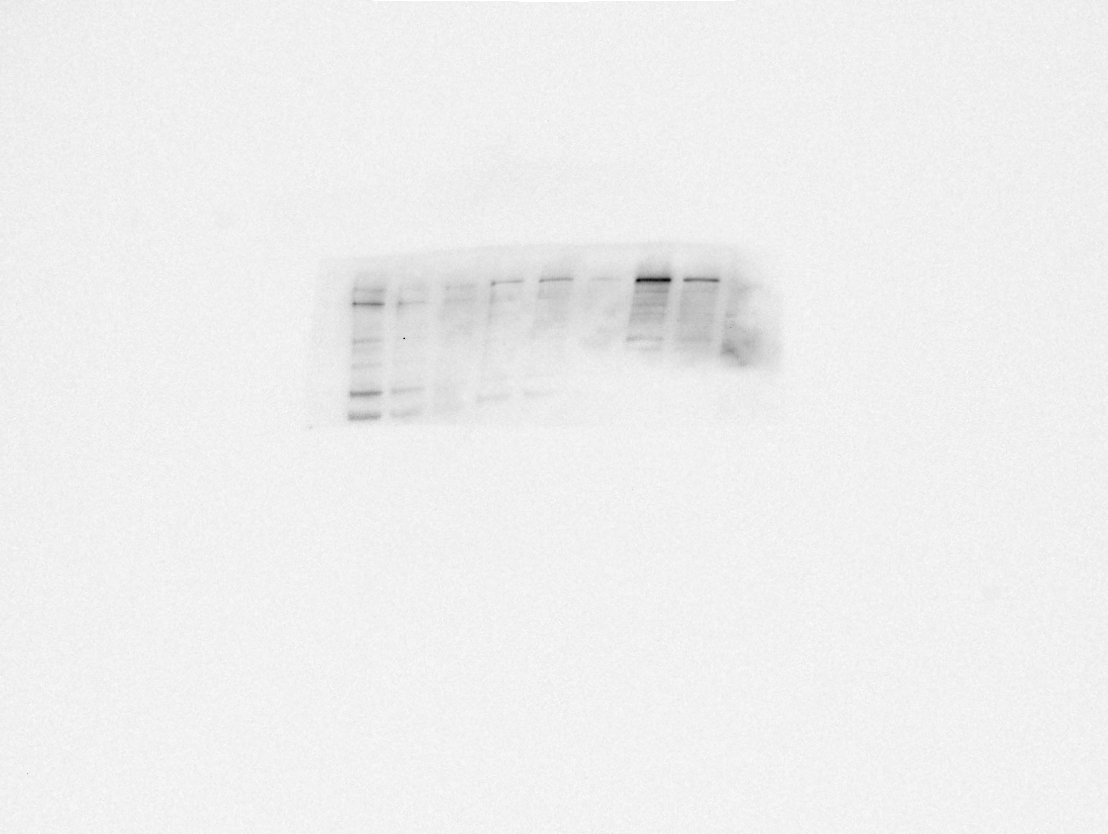

Supplement: Figure 1—source data 1. [file elife-64695-fig1-data1.zip › Fig.1_PanelC_R35X-ATM.jpg]

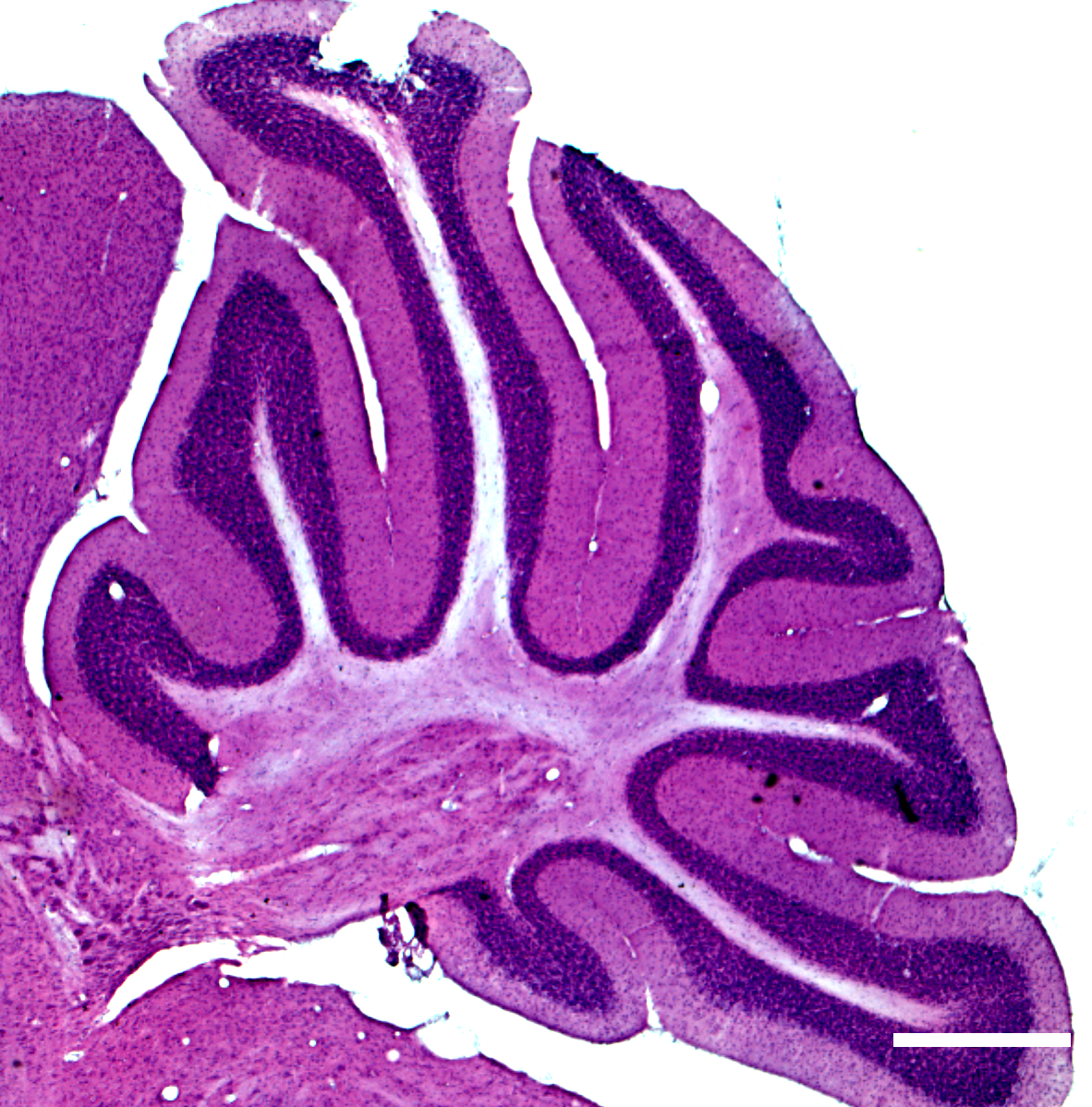

Supplement: Figure 5—source data 1. [file elife-64695-fig5-data1.zip › Figure 5_source data 1/546R_2.5x_S1-2sc.tif]

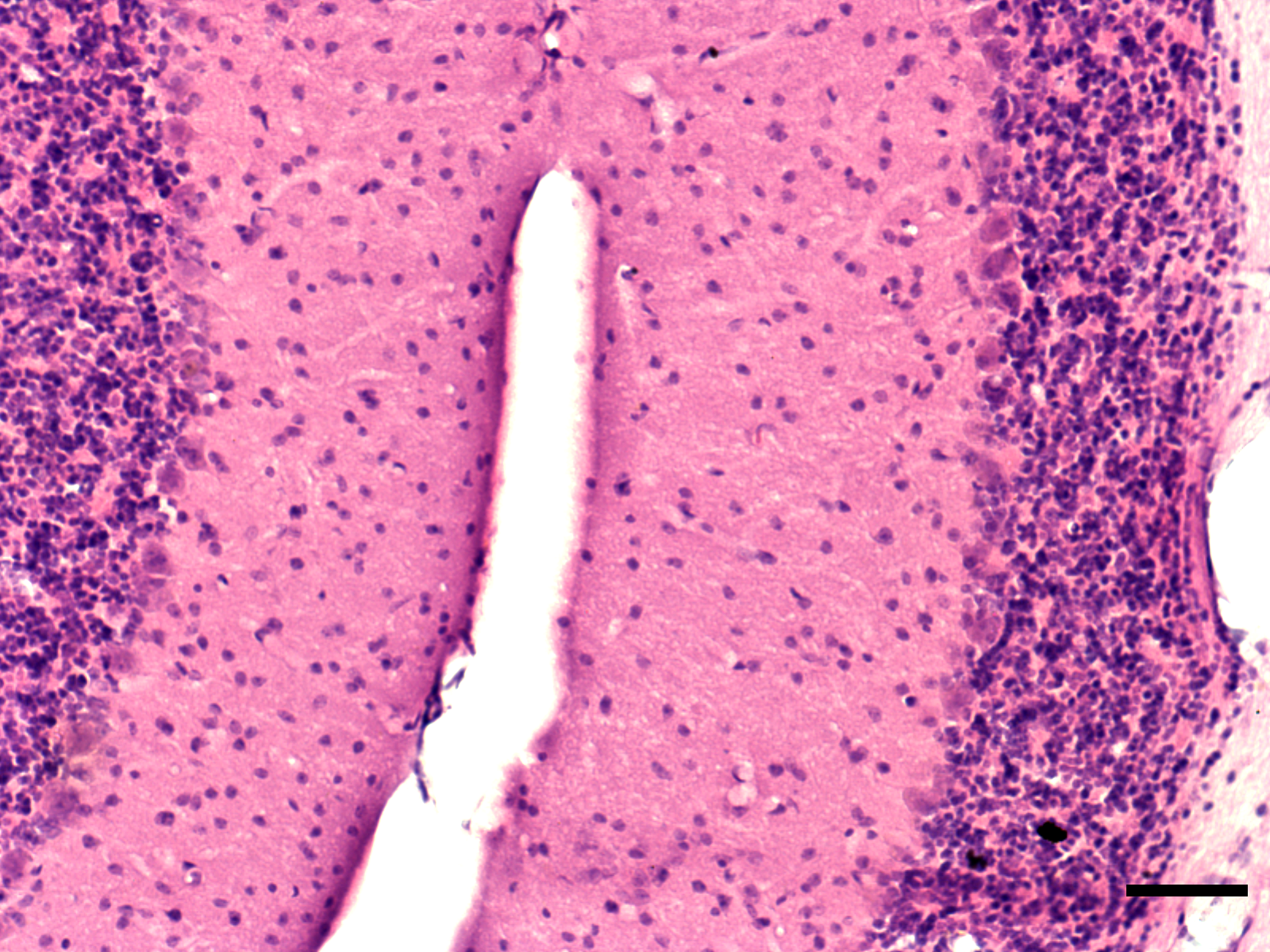

Supplement: Figure 5—source data 1. [file elife-64695-fig5-data1.zip › Figure 5_source data 1/546R_20x_S1-1sc.tif]

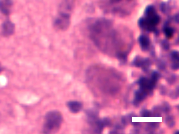

Supplement: Figure 5—source data 1. [file elife-64695-fig5-data1.zip › Figure 5_source data 1/546R_20x_S1-3sc.tif]

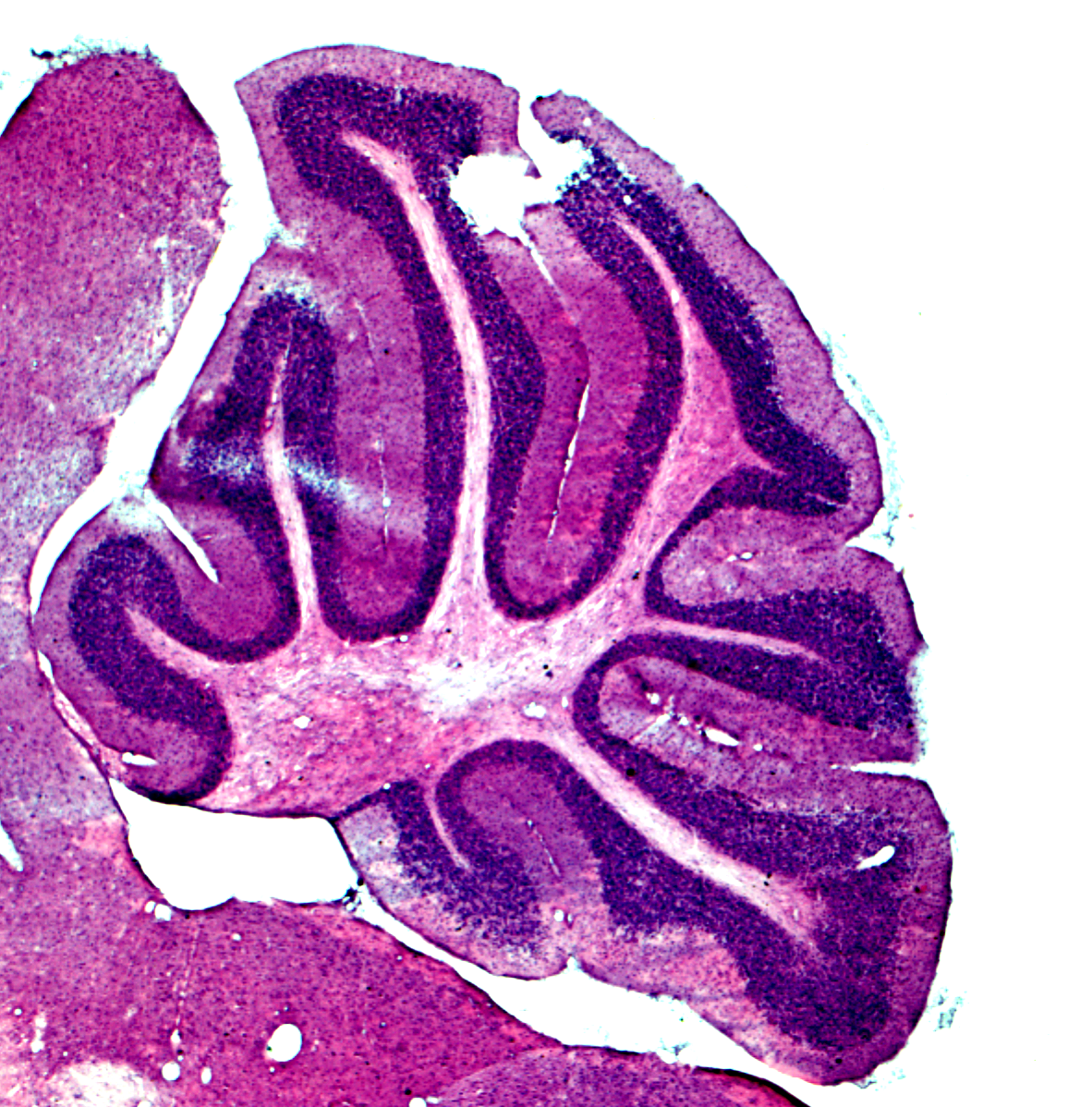

Supplement: Figure 5—source data 1. [file elife-64695-fig5-data1.zip › Figure 5_source data 1/561R_2.5x_S6-2.tif]

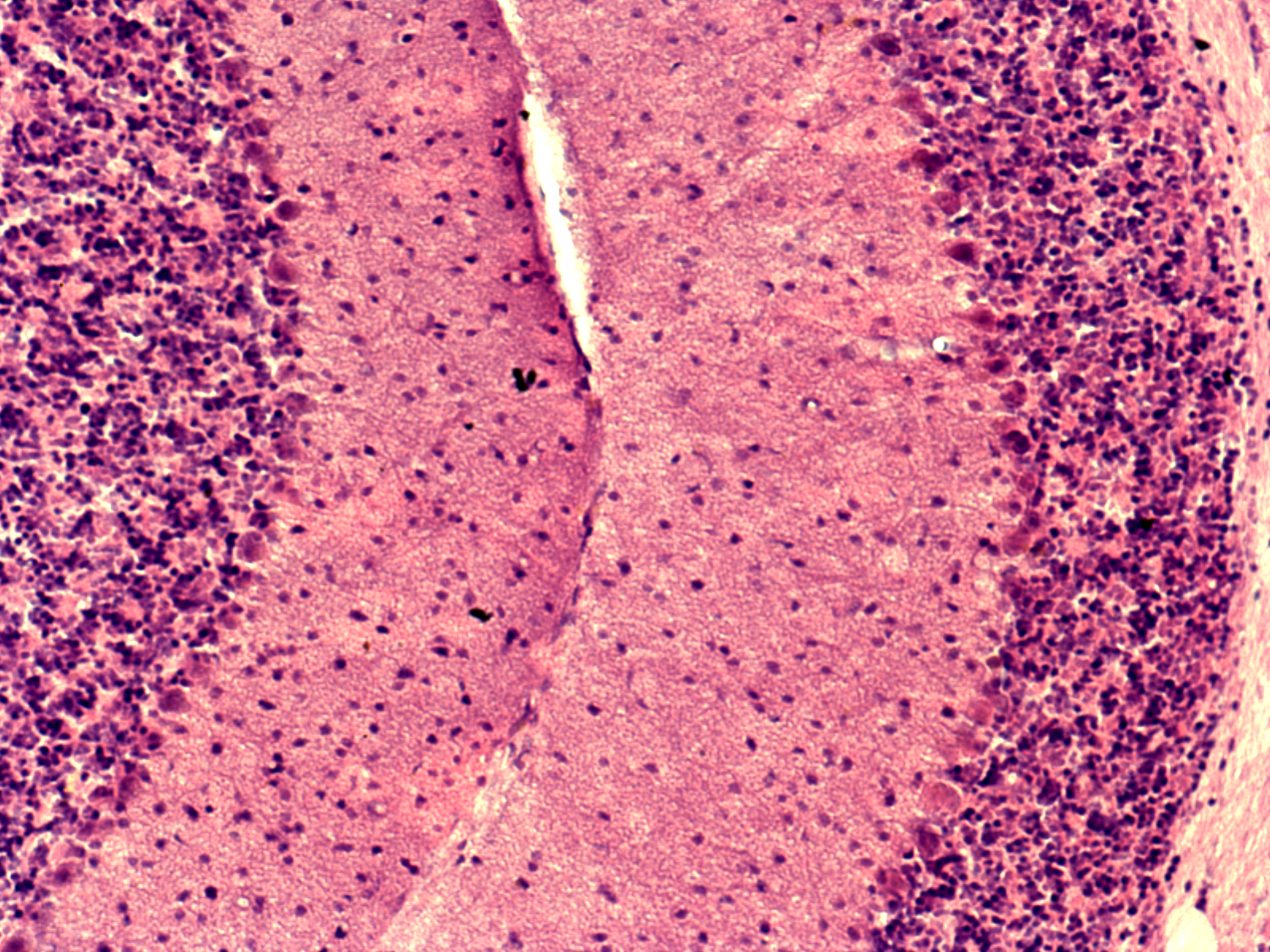

Supplement: Figure 5—source data 1. [file elife-64695-fig5-data1.zip › Figure 5_source data 1/561R_20x_S5-2.tif]

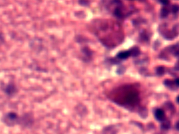

Supplement: Figure 5—source data 1. [file elife-64695-fig5-data1.zip › Figure 5_source data 1/561R_20x_S5-3.tif]

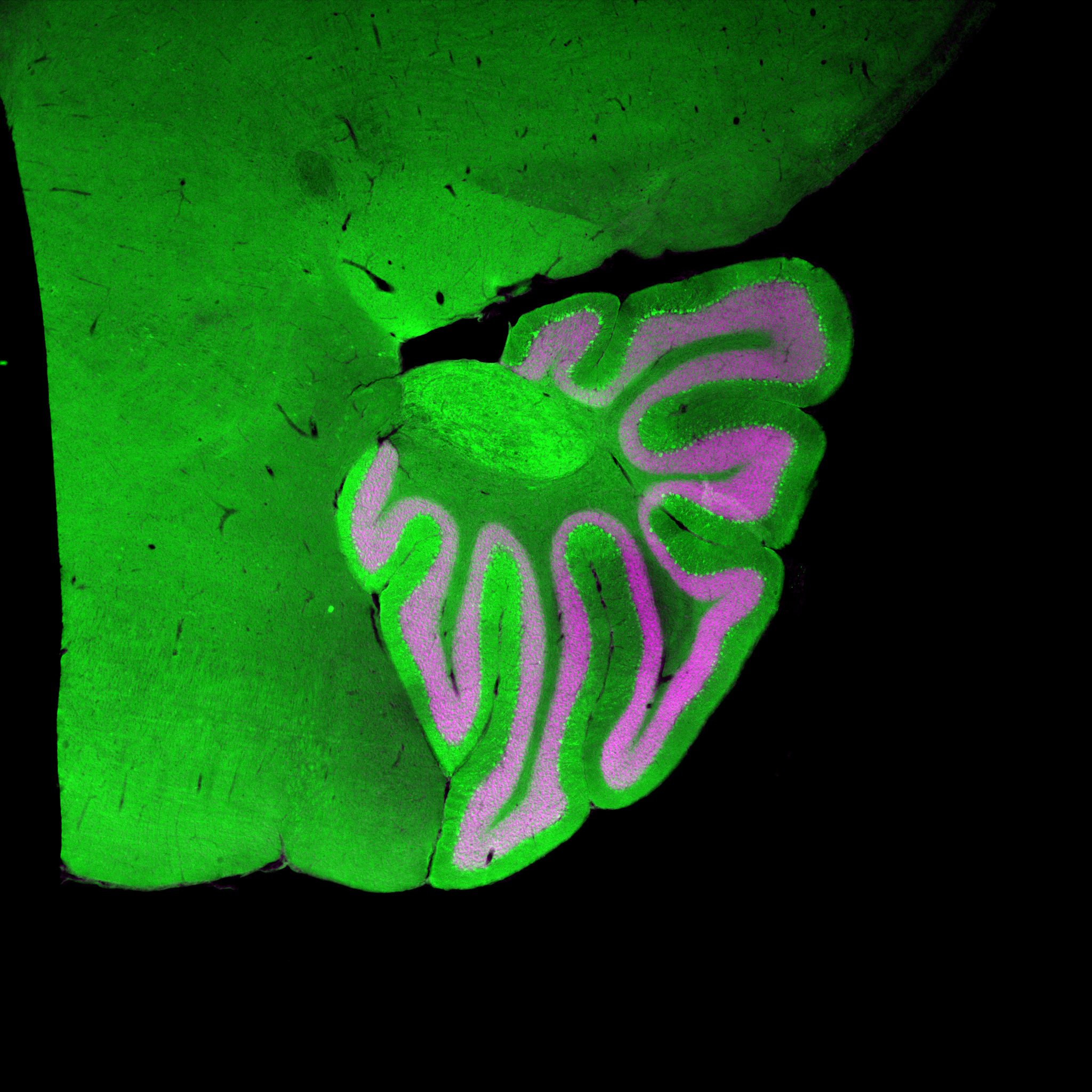

Supplement: Figure 5—source data 1. [file elife-64695-fig5-data1.zip › Figure 5_source data 1/833-Composite (RGB).tif]

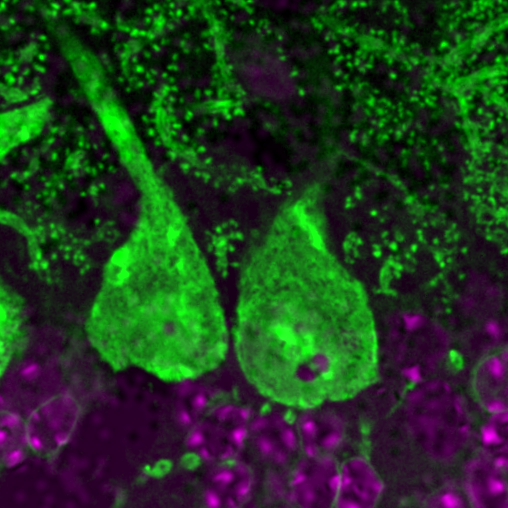

Supplement: Figure 5—source data 1. [file elife-64695-fig5-data1.zip › Figure 5_source data 1/837cr-1.tif]

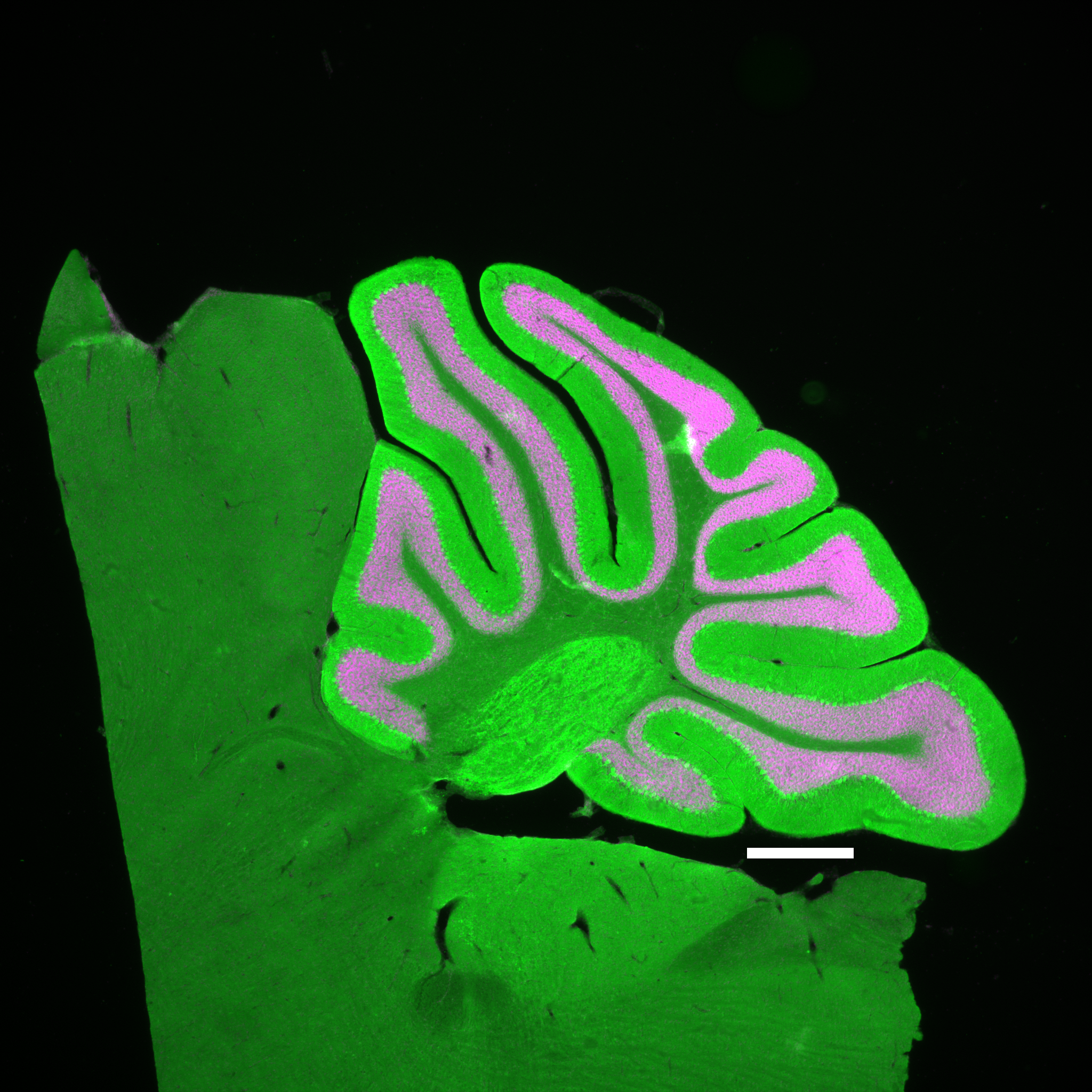

Supplement: Figure 5—source data 1. [file elife-64695-fig5-data1.zip › Figure 5_source data 1/881R-2.5x-slice3-2wscale2.tif]

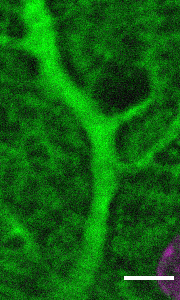

Supplement: Figure 5—source data 1. [file elife-64695-fig5-data1.zip › Figure 5_source data 1/881_63X_lobVIII-5-sb_dendrite-1.tif]

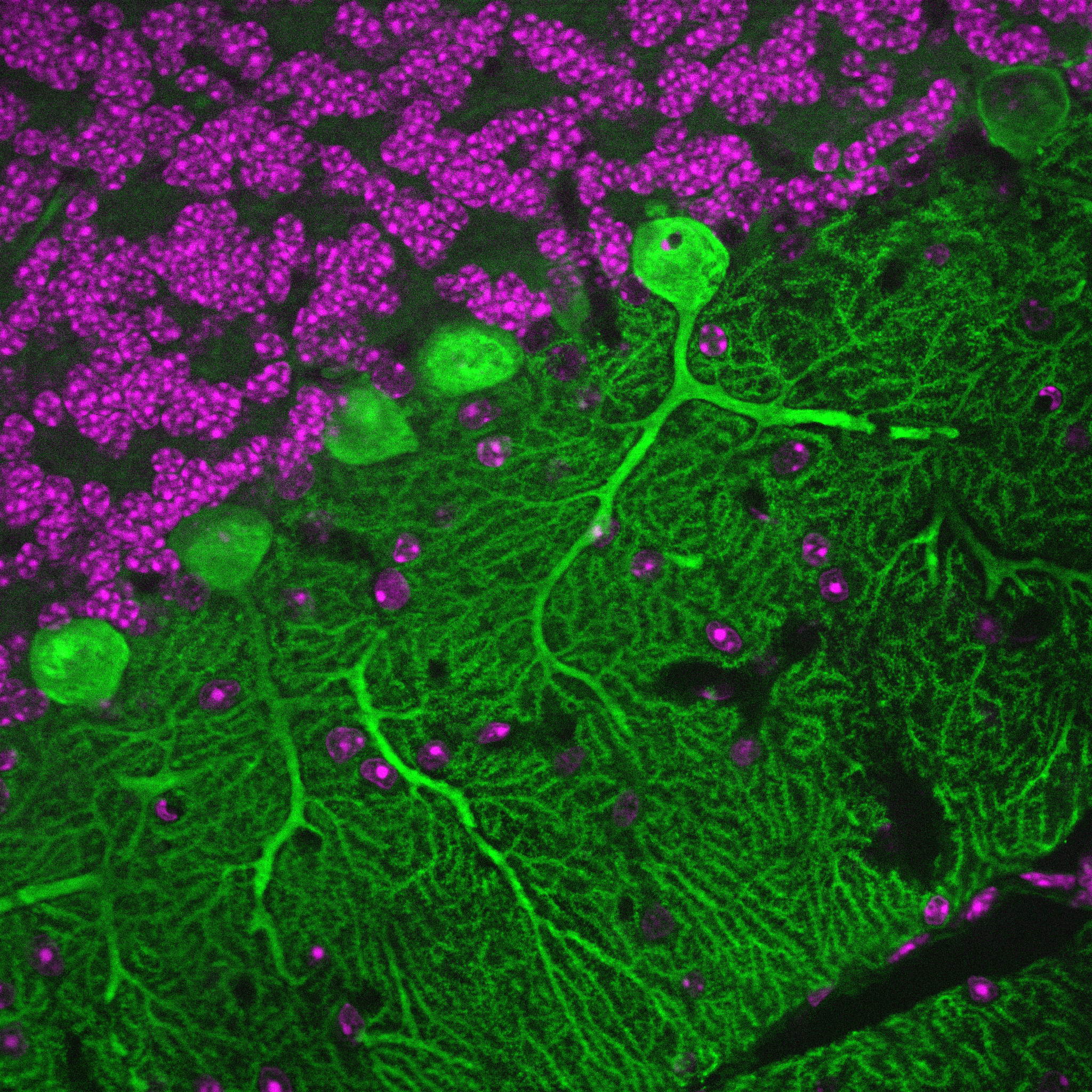

Supplement: Figure 5—source data 1. [file elife-64695-fig5-data1.zip › Figure 5_source data 1/881_63X_lobVIII-5.tif]

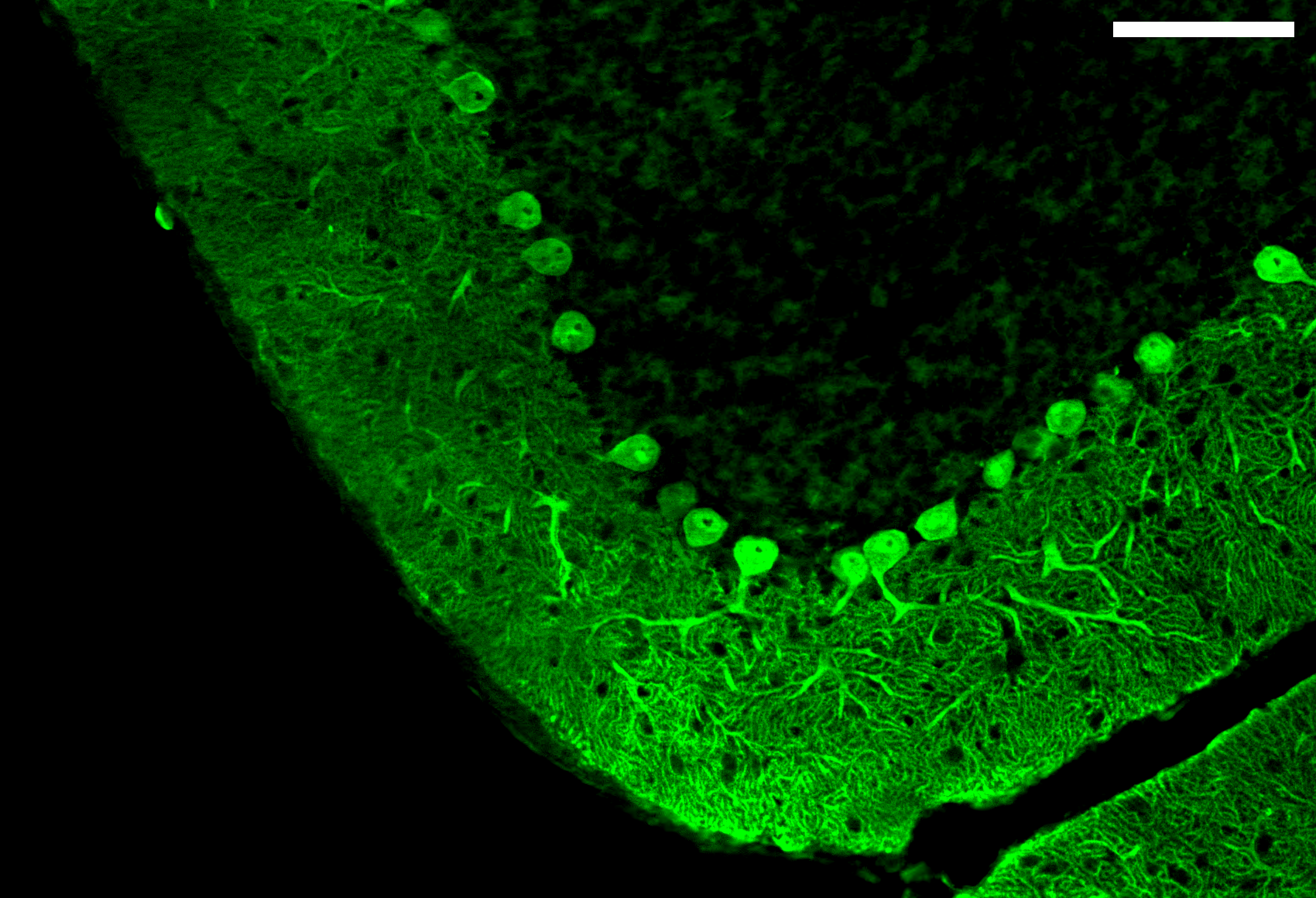

Supplement: Figure 5—source data 1. [file elife-64695-fig5-data1.zip › Figure 5_source data 1/881_lob8R.jp2 #1_2c-2s.tif]

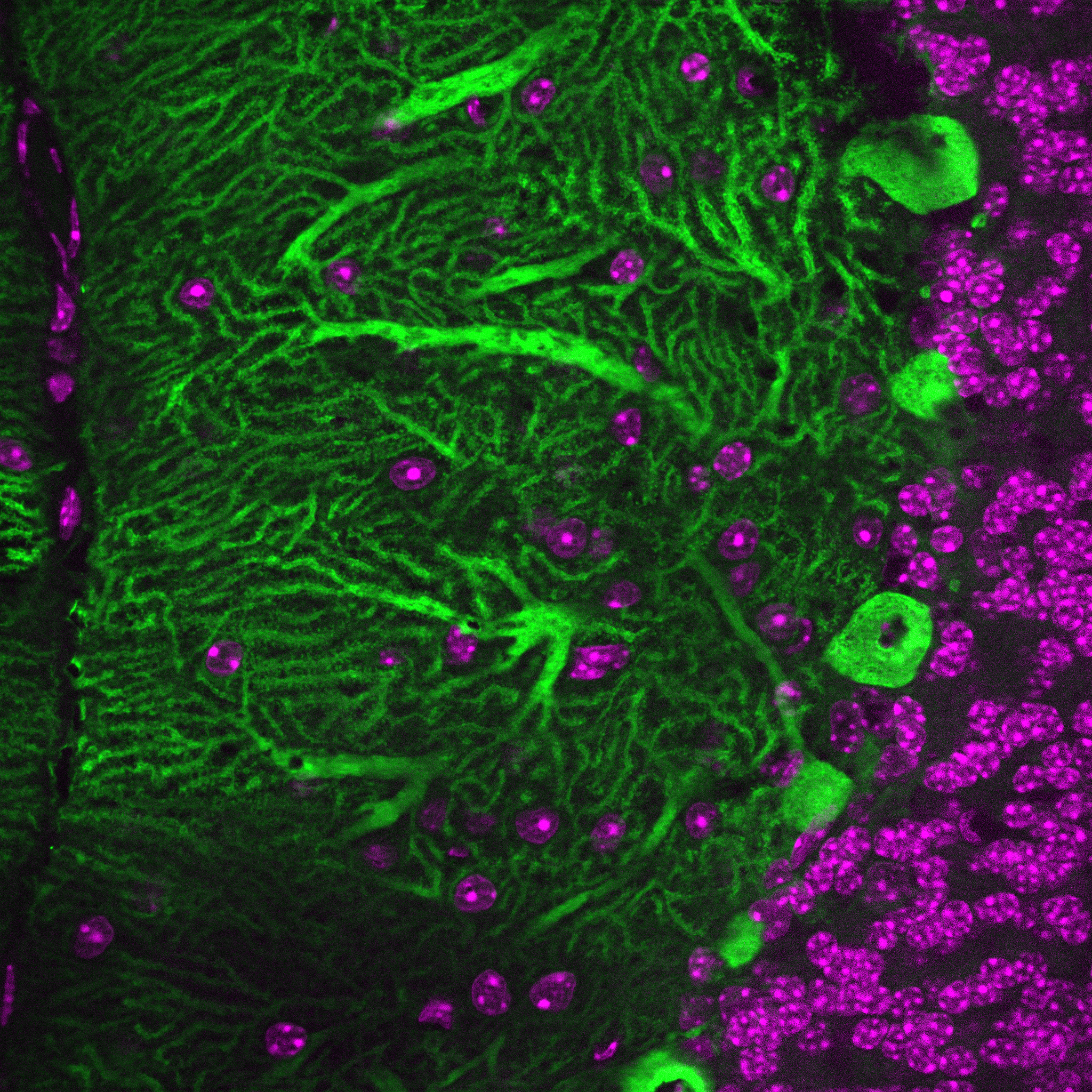

Supplement: Figure 5—source data 1. [file elife-64695-fig5-data1.zip › Figure 5_source data 1/894_63X_lobIV-V-3.tif]

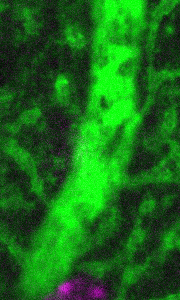

Supplement: Figure 5—source data 1. [file elife-64695-fig5-data1.zip › Figure 5_source data 1/894_63X_lobIV-V-3_dend.tif]

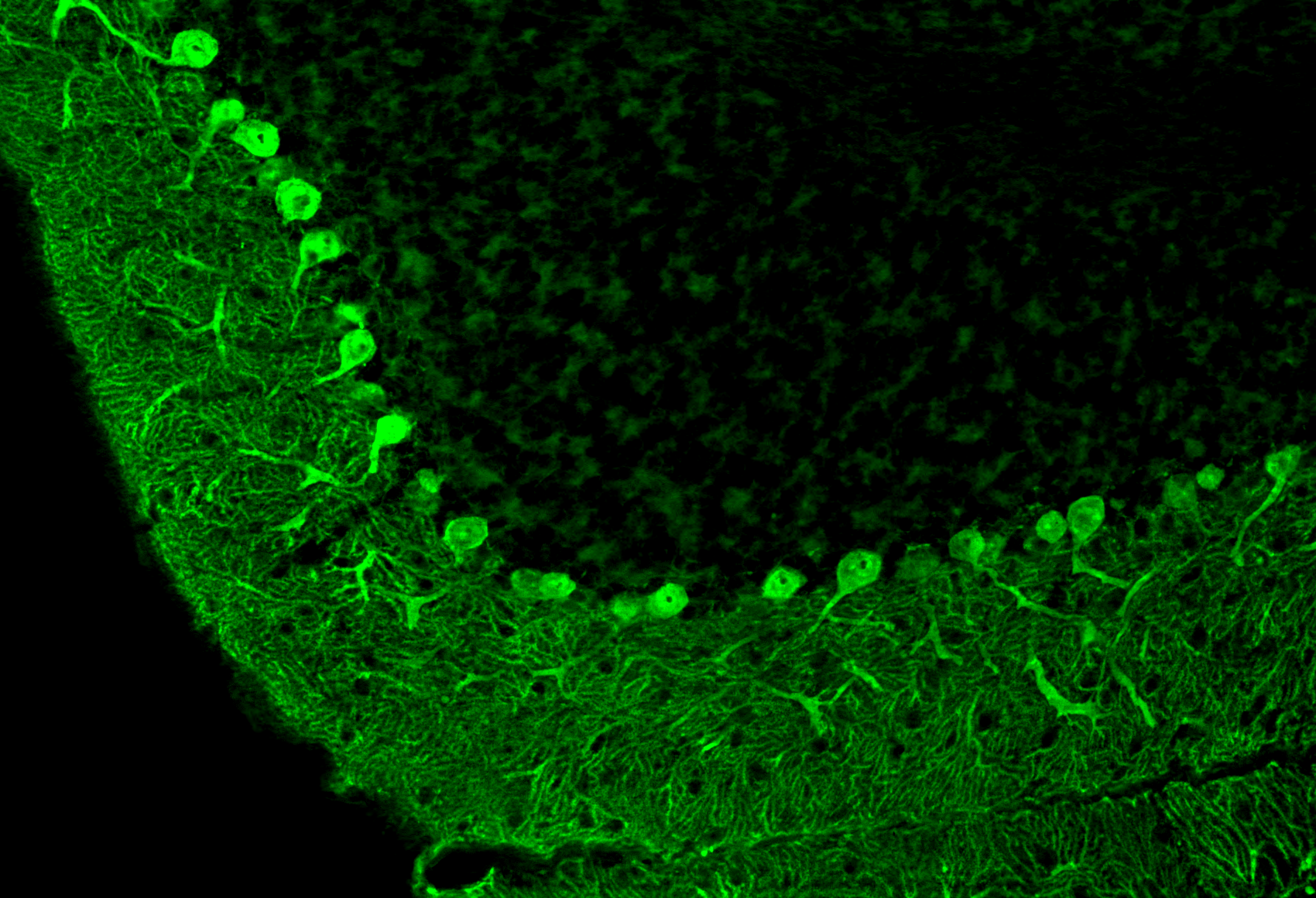

Supplement: Figure 5—source data 1. [file elife-64695-fig5-data1.zip › Figure 5_source data 1/894_lob8R_6.jp2 #1_2c-1.tif]

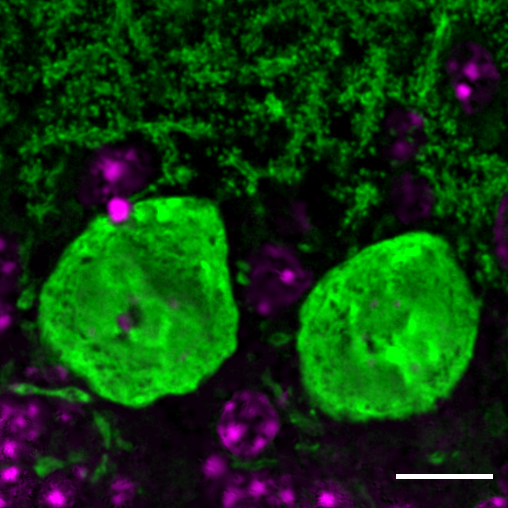

Supplement: Figure 5—source data 1. [file elife-64695-fig5-data1.zip › Figure 5_source data 1/915-sc-cr.tif]

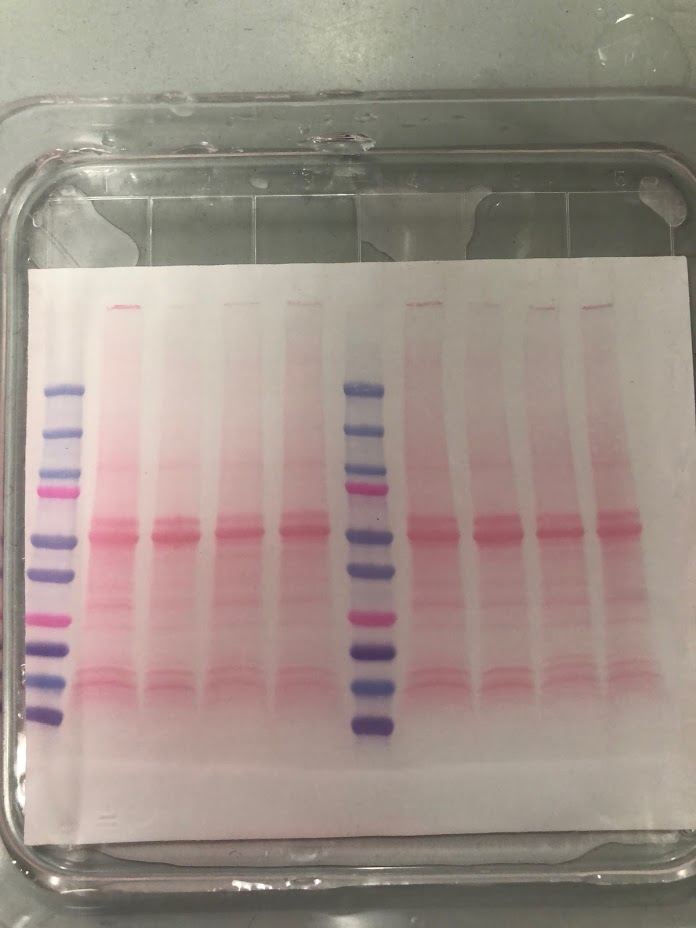

Supplement: Figure 8—source data 1. [file elife-64695-fig8-data1.zip › Fig.8_Panel 3 Ponceau.JPG]

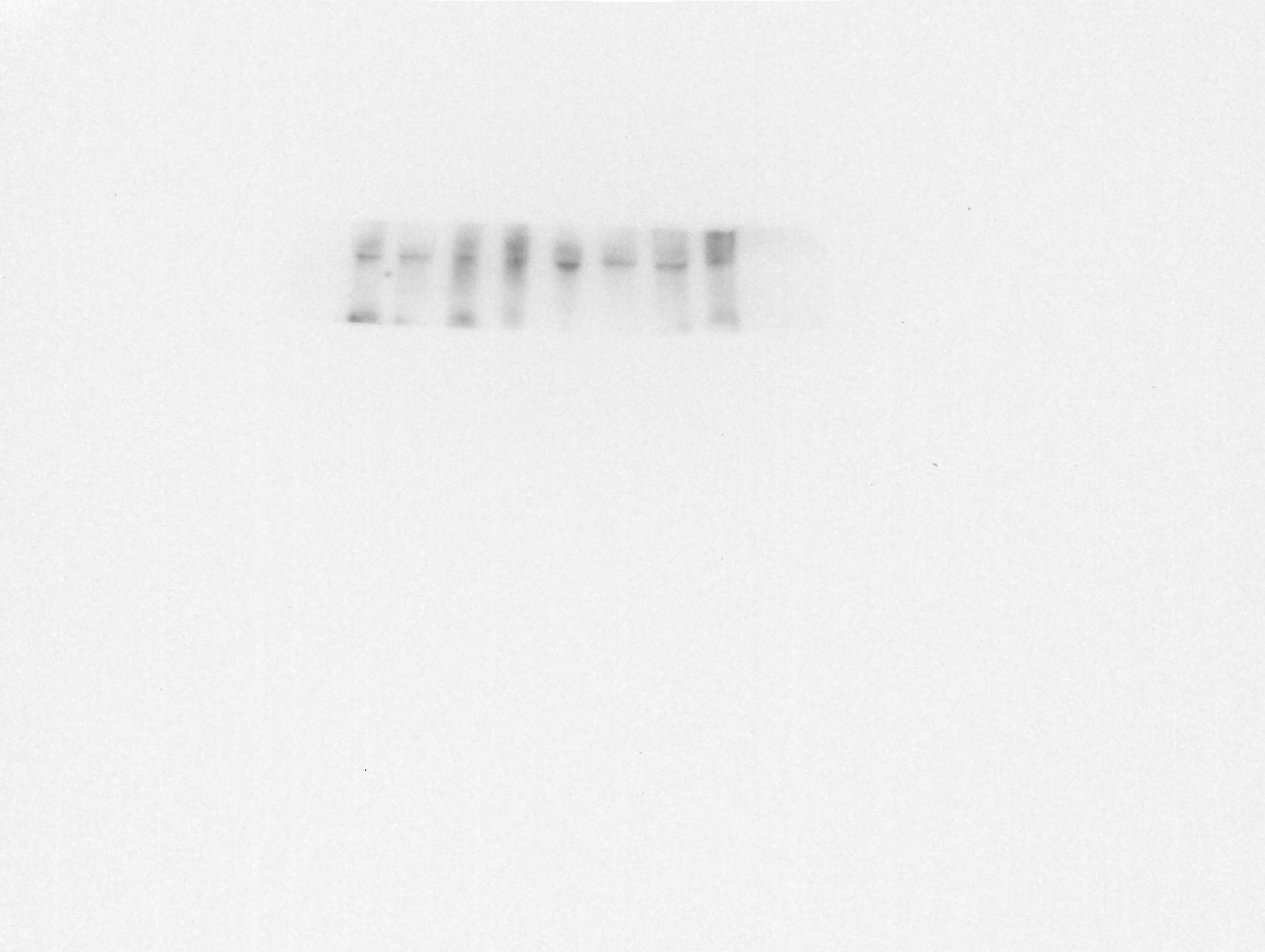

Supplement: Figure 8—source data 1. [file elife-64695-fig8-data1.zip › Fig.8_Panel1_Actin.tif]

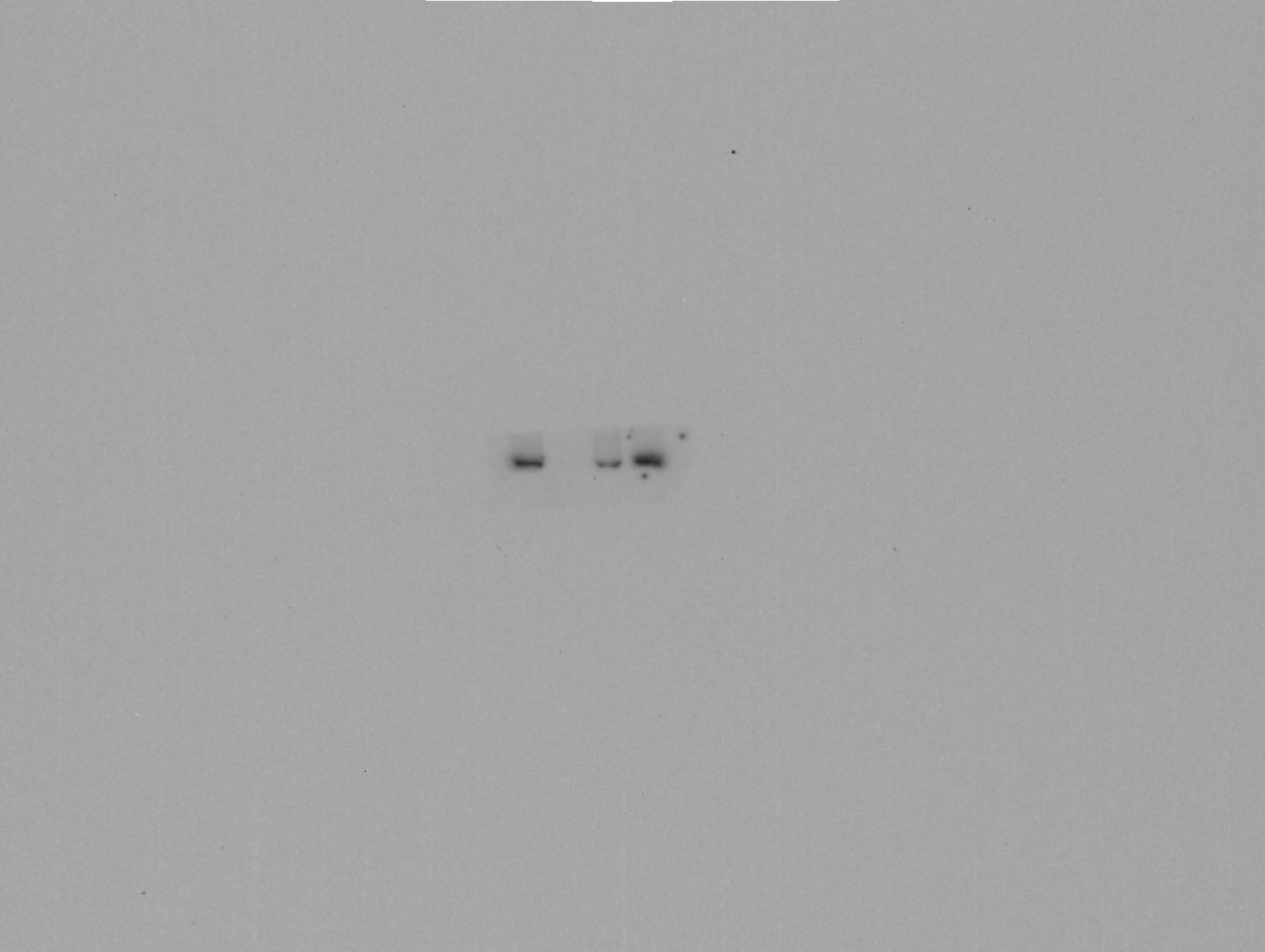

Supplement: Figure 8—source data 1. [file elife-64695-fig8-data1.zip › Fig.8_Panel1_ATM.tif]

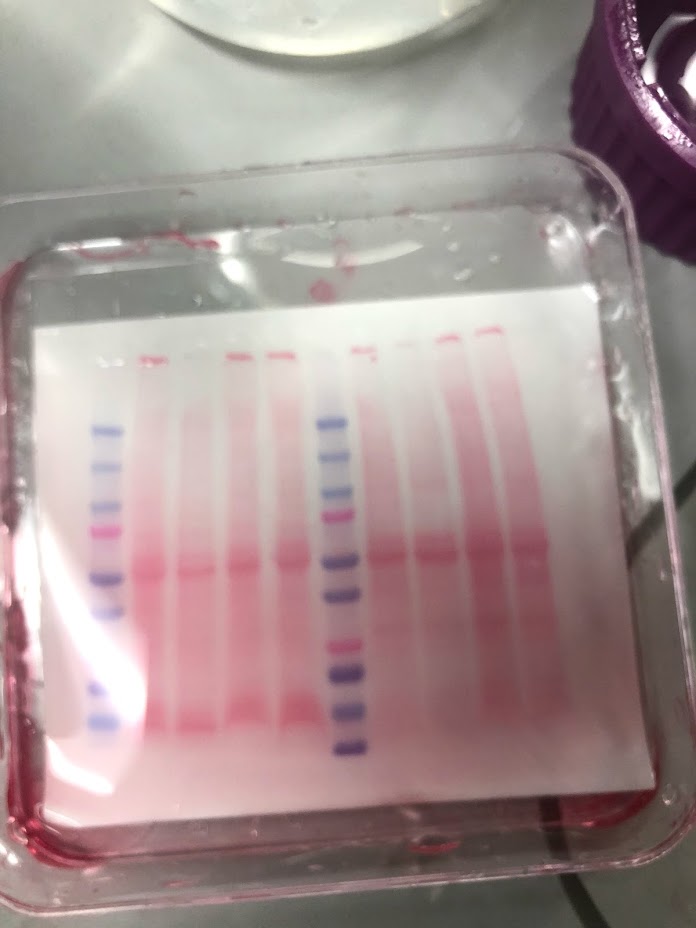

Supplement: Figure 8—source data 1. [file elife-64695-fig8-data1.zip › Fig.8_Panel1_Ponceau.JPG]

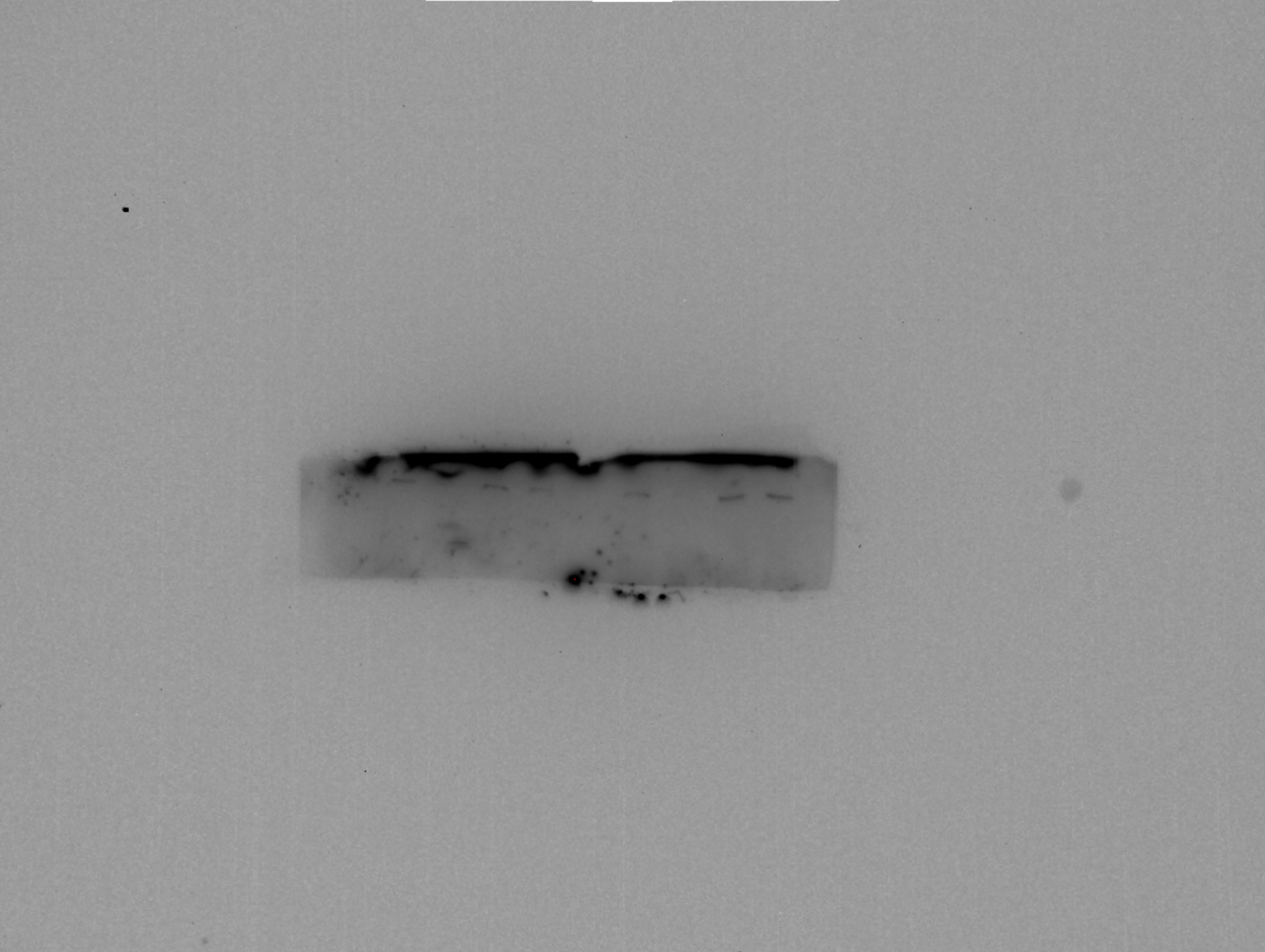

Supplement: Figure 8—source data 1. [file elife-64695-fig8-data1.zip › Fig.8_Panel2_ATM.tif]

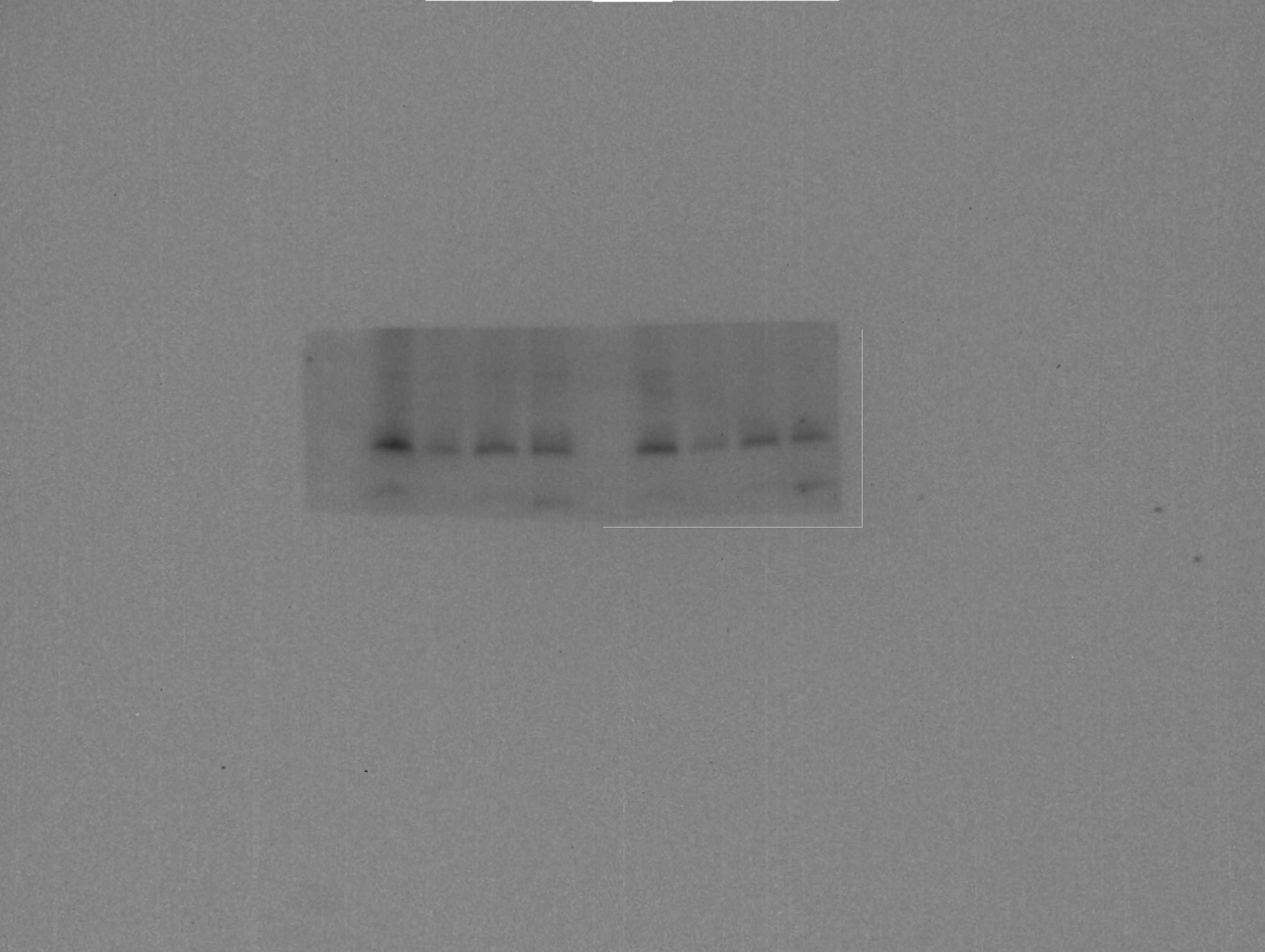

Supplement: Figure 8—source data 1. [file elife-64695-fig8-data1.zip › Fig.8_Panel2_GAPDH.tif]

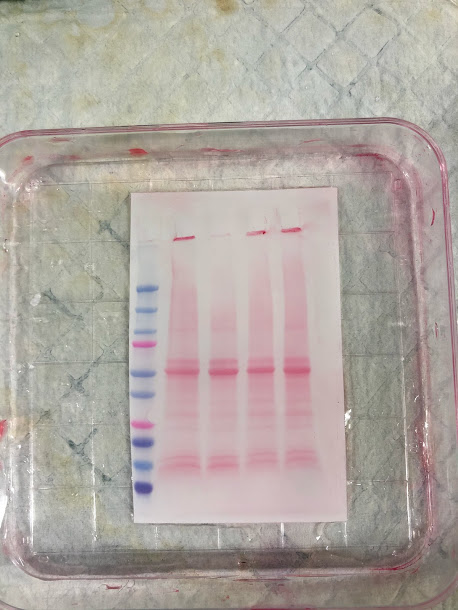

Supplement: Figure 8—source data 1. [file elife-64695-fig8-data1.zip › Fig.8_Panel2_Ponceau.jpg]

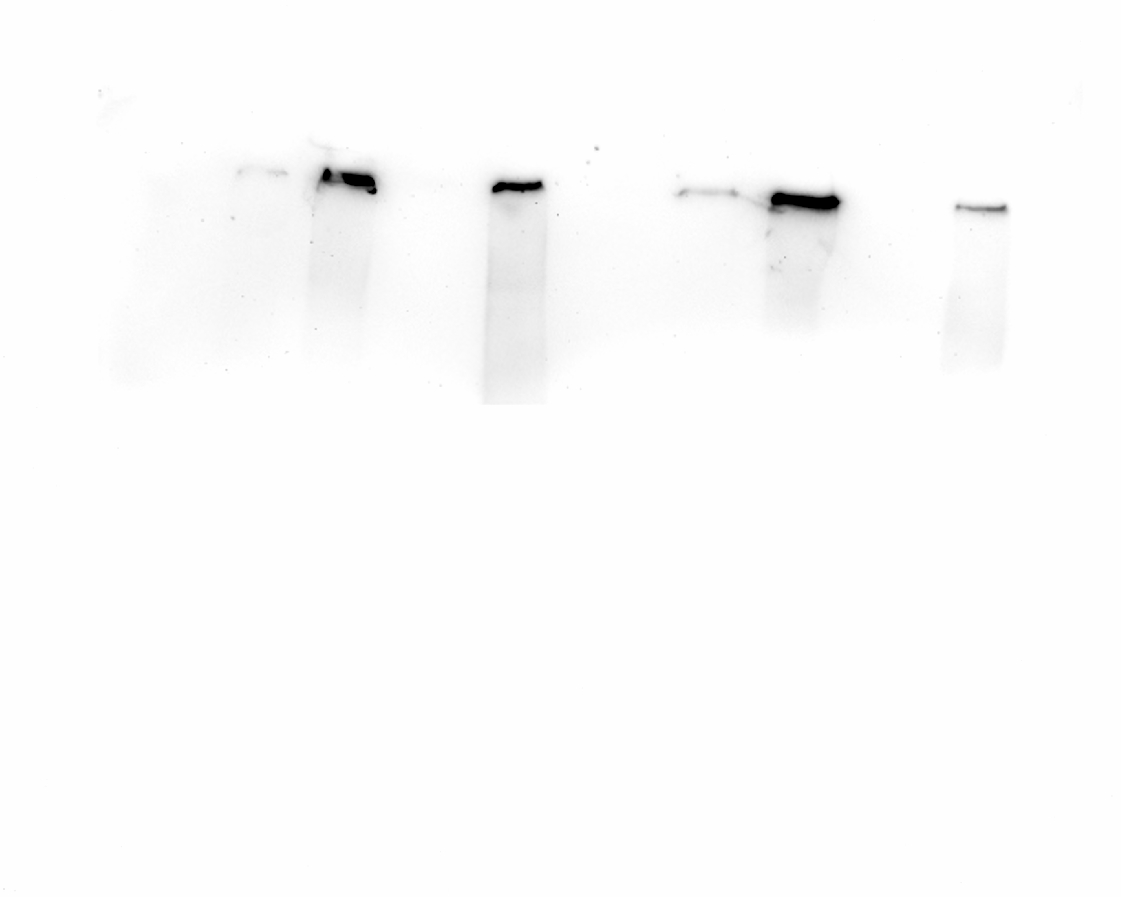

Supplement: Figure 8—source data 1. [file elife-64695-fig8-data1.zip › Fig.8_Panel3_ATM.png]

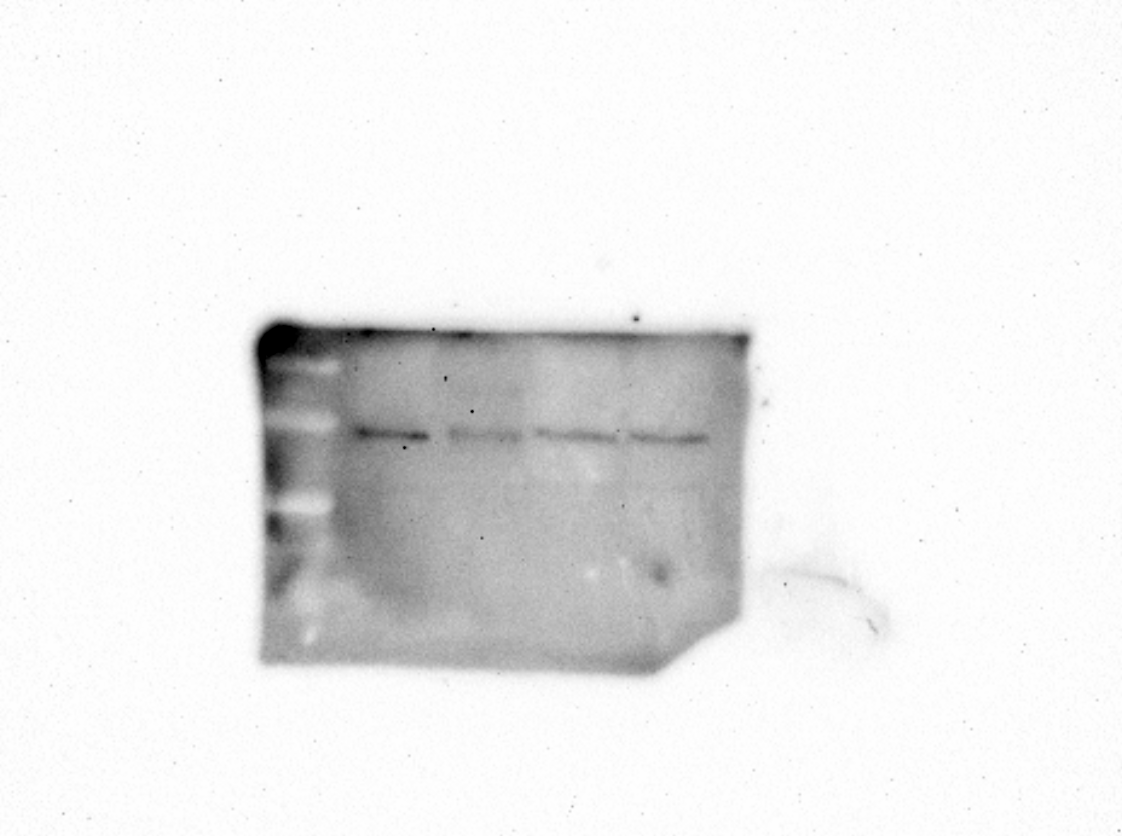

Supplement: Figure 8—source data 1. [file elife-64695-fig8-data1.zip › Fig.8_Panel3_GAPDH.tif]
