# Supplementary material for: A novel, ataxic mouse model of ataxia telangiectasia caused by a clinically relevant nonsense mutation
Source: eLife. 2021 Nov 1;10:e64695. doi: 10.7554/eLife.64695 (PMC8601662; doi:10.7554/eLife.64695)

Panel 1

Panel image

Tech. replicate

1 2 3 4

1) Wildtype
2) $Atm^{R35X/R35X}$
3) $Atm^{R35X/R35X}$ (G418)
4) $Atm^{R35X/R35X}$ (GJ103)

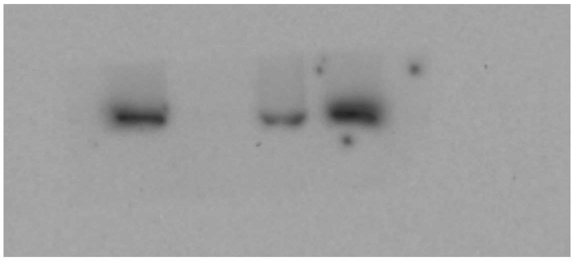

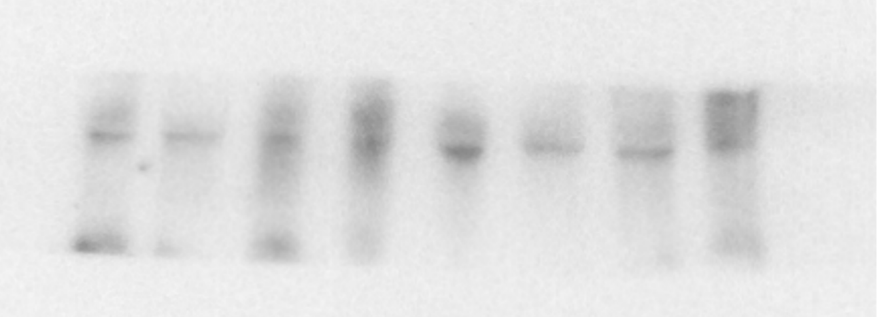

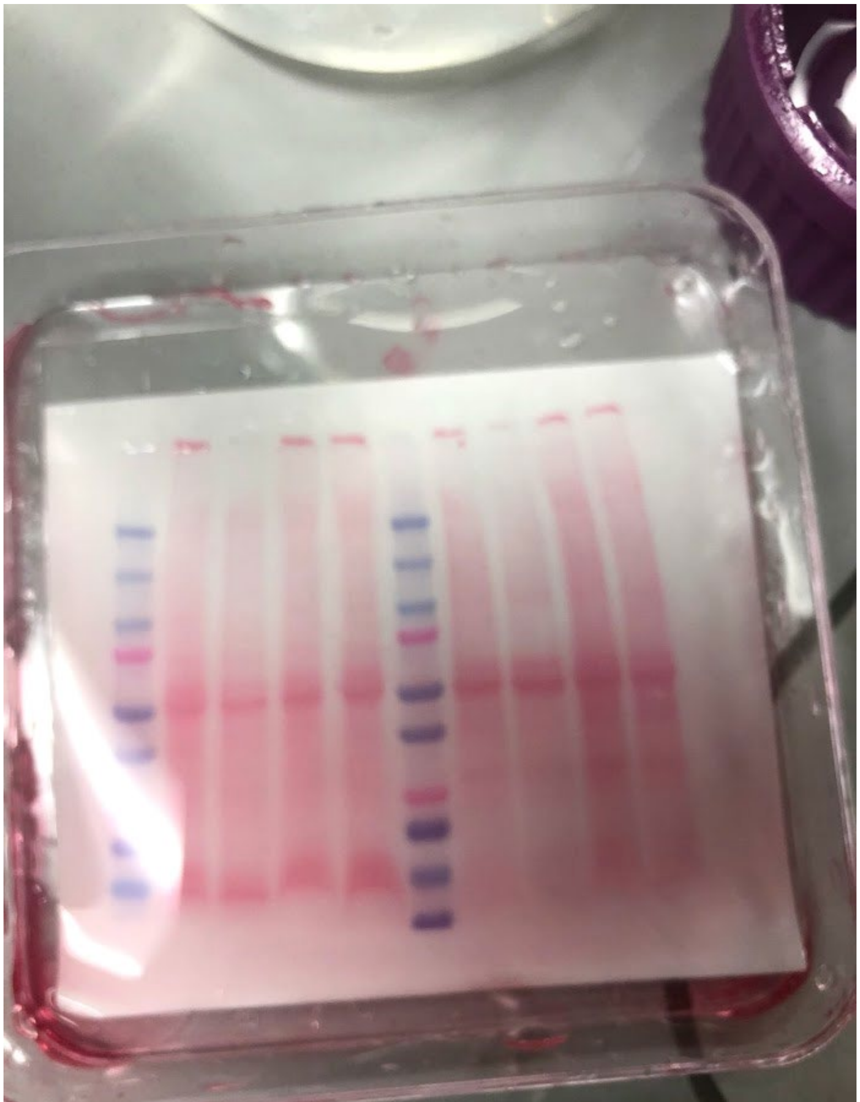

Panel 2

Panel image

Tech. replicate 1 2 3 4

1) Wildtype
2) *$Atm^{R35X/R35X}$*
3) *$Atm^{R35X/R35X}$* (G418)
4) *$Atm^{R35X/R35X}$* (GJ103)

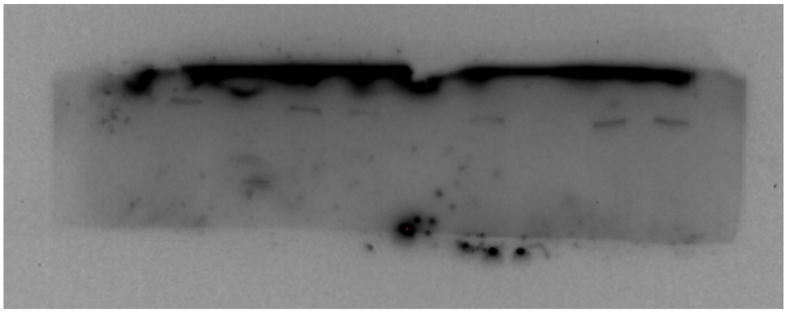

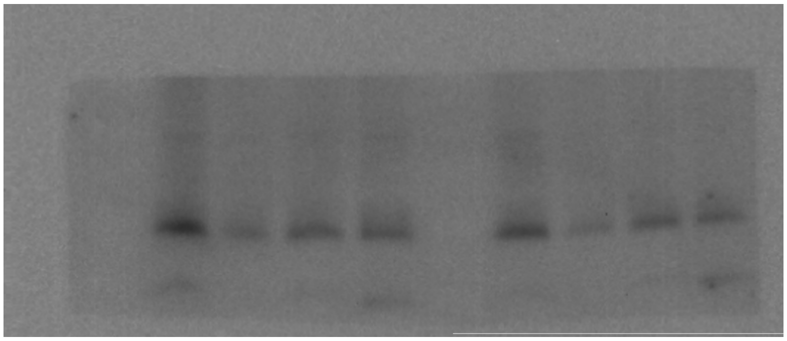

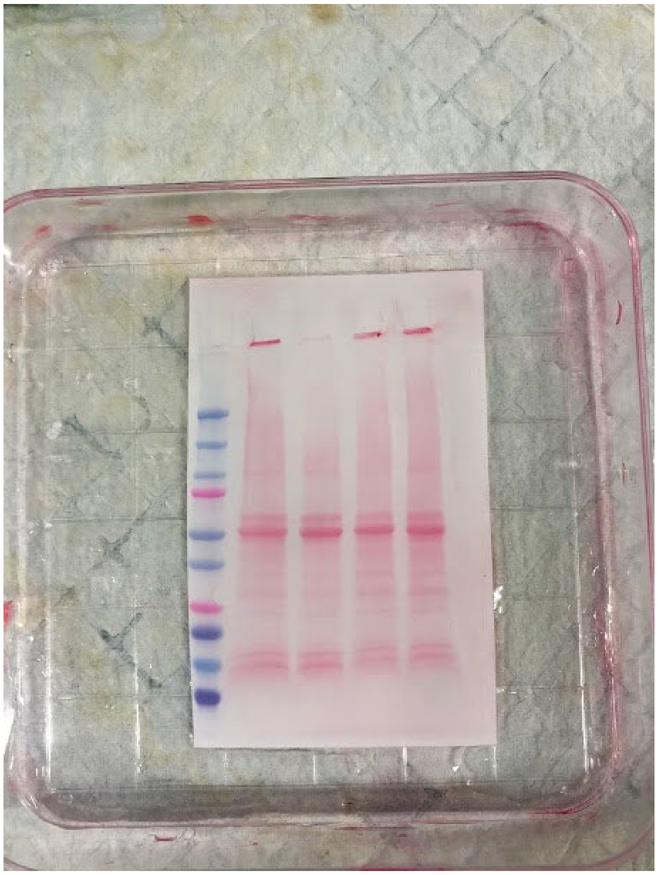

Panel 3

1) Wildtype
2) $Atm^{R35X/R35X}$
3) $Atm^{R35X/R35X}$ (G418)
4) $Atm^{R35X/R35X}$ (GJ103)

Panel image (reversed in figure)

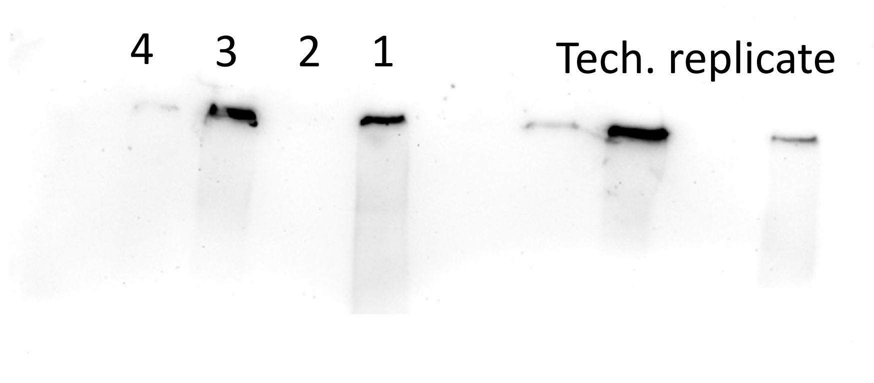

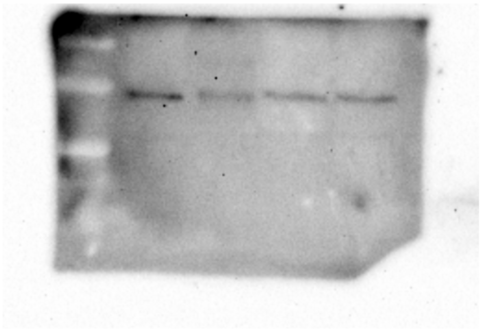

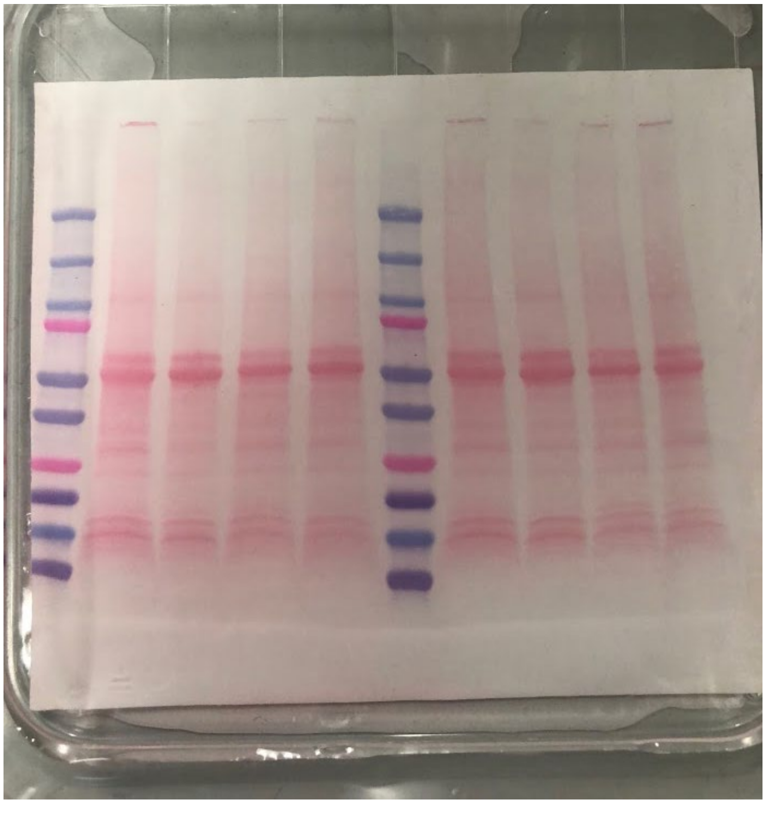

Supplement: Figure 8—source data 1. [file elife-64695-fig8-data1.zip › Fig.8.pdf]
